# TNFR2 Signaling Regulates the Immunomodulatory Function of Oligodendrocyte Precursor Cells

**DOI:** 10.3390/cells10071785

**Published:** 2021-07-15

**Authors:** Haritha L. Desu, Placido Illiano, James S. Choi, Maureen C. Ascona, Han Gao, Jae K. Lee, Roberta Brambilla

**Affiliations:** 1 The Miami Project to Cure Paralysis, Department of Neurological Surgery, University of Miami Miller School of Medicine, Miami, FL 33136, USA; hld32@med.miami.edu (H.L.D.); pxi44@med.miami.edu (P.I.); jsc228@med.miami.edu (J.S.C.); mca47@med.miami.edu (M.C.A.); gaoh35@mail.sysu.edu.cn (H.G.); JLee22@med.miami.edu (J.K.L.); 2 Department of Spine Surgery, The Third Affiliated Hospital of Sun Yat-Sen University, Guangzhou 510630, China; 3 Department of Neurobiology Research, Institute of Molecular Medicine, and BRIDGE—Brain Research Inter Disciplinary Guided Excellence, 5000 Odense, Denmark; 4 Department of Clinical Research, University of Southern Denmark, 5000 Odense, Denmark

**Keywords:** oligodendrocytes, neuroinflammation, remyelination, cytokines, TNF, multiple sclerosis

## Abstract

Multiple sclerosis (MS) is a neuroimmune disorder characterized by inflammation, CNS demyelination, and progressive neurodegeneration. Chronic MS patients exhibit impaired remyelination capacity, partly due to the changes that oligodendrocyte precursor cells (OPCs) undergo in response to the MS lesion environment. The cytokine tumor necrosis factor (TNF) is present in the MS-affected CNS and has been implicated in disease pathophysiology. Of the two active forms of TNF, transmembrane (tmTNF) and soluble (solTNF), tmTNF signals via TNFR2 mediating protective and reparative effects, including remyelination, whereas solTNF signals predominantly via TNFR1 promoting neurotoxicity. To better understand the mechanisms underlying repair failure in MS, we investigated the cellular responses of OPCs to inflammatory exposure and the specific role of TNFR2 signaling in their modulation. Following treatment of cultured OPCs with IFNγ, IL1β, and TNF, we observed, by RNA sequencing, marked inflammatory and immune activation of OPCs, accompanied by metabolic changes and dysregulation of their proliferation and differentiation programming. We also established the high likelihood of cell–cell interaction between OPCs and microglia in neuroinflammatory conditions, with OPCs able to produce chemokines that can recruit and activate microglia. Importantly, we showed that these functions are exacerbated when TNFR2 is ablated. Together, our data indicate that neuroinflammation leads OPCs to shift towards an immunomodulatory phenotype while diminishing their capacity to proliferate and differentiate, thus impairing their repair function. Furthermore, we demonstrated that TNFR2 plays a key role in this process, suggesting that boosting TNFR2 activation or its downstream signals could be an effective strategy to restore OPC reparative capacity in demyelinating disease.

## 1. Introduction

Multiple sclerosis (MS), a chronic autoimmune neuroinflammatory disorder, is the leading cause of neurological disability in young adults [1]. One of the hallmarks of MS is oligodendrocyte death that results in the formation of demyelinating lesions within the CNS, both in the white and gray matter [2]. Active lesions, which are prevalent in the acute phase of relapsing-remitting MS (RRMS), are characterized by the presence of immune cell infiltrates and high levels of inflammatory factors, including cytokines and chemokines [3,4]. The efficacy of approved disease modifying therapies (DMT) for RRMS is in fact due, predominantly, to their ability to reduce this immune-inflammatory burden [5]. DMTs, however, do not promote remyelination, the key process necessary for lesion repair, or neuroprotection and, thus, are ineffective in progressive MS forms wherein axonal damage and neurodegeneration are prominent pathological correlates [6]. Lesion repair failure has been attributed to the compromised differentiation capacity of oligodendrocyte precursor cells (OPCs) in individuals with MS [7]. Although this has been suggested to depend on the neurotoxic lesion environment, the mechanisms that drive OPC fate in MS and underlie remyelination impairment have yet to be fully understood. 

Tumor necrosis factor (TNF) is a pleiotropic cytokine that has been linked to MS pathophysiology [8]. Indeed, TNF is found at high levels in the serum, cerebrospinal fluid, and active lesions of individuals with MS [9,10,11,12], correlating with lesion severity and disease progression [10]. TNF exists in two active forms: a native transmembrane form, tmTNF, which functions via cell-to-cell contact, and a soluble form, solTNF, generated via the enzymatic cleavage of tmTNF [13] by the metalloproteinase ADAM17 [14,15]. SolTNF preferentially binds to and activates TNFR1, whereas tmTNF, which can bind to both receptors, is the main activating ligand of TNFR2 [16,17]. Signaling downstream of TNFR1 and TNFR2 often results in opposing effects during disease progression, with TNFR1 typically initiating cell death and chronic inflammatory mechanisms via canonical NF-κB activation and TNFR2 promoting pro-survival and reparative cascades via the activation of PI3K/AKT and non-canonical NF-κB signaling [18].

Several reports, including our own, have suggested that activation of TNFR2 signaling in oligodendroglia is beneficial in MS. A seminal study by Arnett et al. using germline TNFR2 knockout mice in the cuprizone model of demyelination demonstrated that TNFR2 is essential for oligodendrocyte differentiation and remyelination [19]. At our end, using a conditional knockout model where TNFR2 was specifically ablated throughout the oligodendrocyte lineage (CNPcre:TNFR2^fl/fl^ mice), we showed that TNFR2 is implicated not only in the differentiation of oligodendrocytes but also in their immunomodulatory function [20,21]. Indeed, induced with experimental autoimmune encephalomyelitis (EAE), CNPcre:TNFR2^fl/fl^ mice showed earlier disease onset and chronic EAE exacerbation, which correlated with increased axonal damage and impaired remyelination. The early disease onset was preceded by accelerated immune cell infiltration, expression of proinflammatory cytokines, and microglial activation in the spinal cord [20]. Together, these findings pointed at a role for TNFR2 in suppressing the inflammatory and immunomodulatory function of oligodendroglia during neuroimmune disease. Furthermore, *in vitro* data suggested that it is TNFR2 signaling in OPCs, rather than in mature oligodendrocytes (OLs), to be primarily implicated in these processes [20]. 

Based on this collective evidence, to better understand the mechanisms underlying repair failure in MS, we investigated the cellular responses of OPCs to inflammatory activation and the specific role of TNFR2 in their modulation. Following the treatment of cultured OPCs with IFNγ, IL1β, and TNF, cytokines that are highly expressed in the MS lesion environment, we assessed transcriptional changes by RNA sequencing (RNAseq) and observed a marked shift towards an immunomodulatory phenotype, as well as metabolic alterations that could increase vulnerability to cell stressors. We also observed that gene signatures associated with proliferation and differentiation were affected in OPCs, pointing at a potential reduction in their repair capacity. Through bioinformatics, we mapped putative ligand–receptor interactions between OPCs and microglia, indicating the high likelihood that OPC-produced chemokines act on microglia, enhancing their activation state and migration. Notably, these outcomes were exacerbated in OPCs lacking TNFR2, which exhibited further metabolic dysregulation and upregulation of inflammatory processes, specifically of molecules known to promote microglia proliferation and immune-cell migration. 

In summary, we showed that OPCs shift towards an immunomodulatory phenotype when exposed to a neuroinflammatory environment and TNFR2 is implicated in the regulation of this process. Our data indicate that the activation of TNFR2 signaling may redirect OPCs towards proliferation, differentiation, and repair by suppressing their immunomodulatory and inflammatory function, underscoring the relevance of TNFR2 as a target for CNS repair. 

## 2. Materials and Methods

### 2.1. Mice

Germline TNFR2^−/−^ (stock #002620) and WT C57BL/6J mice were obtained from the Jackson Laboratory and used to generate primary cell cultures. All mice were group-housed (maximum 5 mice/cage) in the Animal Core Facility of The Miami Project to Cure Paralysis in a virus-/antigen-free, temperature and humidity controlled room with a 12 h light/dark cycle and free access to water and food. All experiments were performed according to protocols and guidelines approved by the Institutional Animal Care and Use Committee of the University of Miami.

### 2.2. Primary Oligodendrocyte Cultures and Cytokine Stimulation

Cortices from postnatal day 3–5 pups were dissected out and dissociated into single cell suspensions with the Papain Neural Tissue Dissociation Kit (Miltenyi Biotec). Oligodendrocyte precursor cells (OPCs) were isolated by MACS separation using LS columns (Miltenyi Biotec) after incubation with anti-PDGFRα conjugated magnetic microbeads (Miltenyi Biotec). PDGFRα^+^ cells were seeded on poly-d-lysine/laminin coated 24-well plates (40,000 cells/well) and maintained in OPC medium consisting of DMEM/F12 supplemented with 1% N2, 2% B27, 0.01% BSA, 1% penicillin/streptomycin, 10 ng/mL PDGF-AA, and 10 ng/mL FGF2. Cells were cultured for 3 days, with medium replacement (half the volume) every other day. After 3 days *in vitro* (DIV), OPCs were stimulated with a cytokine cocktail of recombinant mouse TNF, IFNγ, and IL1β (all at a concentration of 25 ng/mL, BioLegend) or exposed to the PBS vehicle, then assessed for: (1) intracellular signaling activation: OPCs stimulated for 10 and 20 min, then collected for western blot; (2) differential gene expression: OPCs stimulated for 3 h, then collected for RNA sequencing (RNAseq). In select experiments, OPCs were stimulated with a cytokine cocktail or PBS for 3 h then switched to oligodendrocyte (OL) differentiation medium consisting of DMEM/F12 supplemented with 1% N2, 2% B27, 0.01% BSA, 1% penicillin/streptomycin, 10 ng/mL CNTF, and 40 ng/mL T3. Cells were differentiated to pre-myelinating OLs for 4 days, with medium replacement (half the volume) every other day, then fixed. 

### 2.3. Immunocytochemistry

Cells were fixed with 4% paraformaldehyde (PFA), blocked with 5% normal goat serum, and incubated with antibodies against PDGFRα (rat, 1:200; #558774, BD Pharmingen), Olig2 (rabbit, 1:500; #AB9610, Millipore), and MBP (rat, 1:500; #MAB386, Millipore). For the immunolabeling of O4^+^ OLs, live cells were incubated with O4 hybridoma cell supernatant (kindly provided by Dr. Paula Monje) prior to fixation. Immunoreactivity was visualized with secondary species-specific fluorescent antibodies (1:750; Alexa Fluor-594 and Alexa Fluor-488, Invitrogen). Representative images were taken with a Zeiss Axiovert A1 fluorescence microscope. Cells stained with Olig2, O4, and MBP were analyzed (cell numbers and morphology) in an unbiased manner from 9 randomly selected fields of view per biological replicate at 10× magnification using the Cellomics ArrayScan VTI high-content analysis system (ThermoScientific, Waltham, MA, USA). 

### 2.4. Flow Cytometry

OPCs were isolated, cultured, and stimulated as described above, then switched to a cytokine-free medium overnight (approximately 18 h). Cell were then collected after 5 min incubation with accutase, spun down and resuspended in live/dead staining solution (GhostDye Vio540, Tonbo Biosciences). After 30 min at 4 °C, cells were resuspended in 100 μL flow cytometry buffer (FACS buffer, eBioscience), blocked with anti-CD16/32 (FCR block, eBioscience) for 5 min at RT, and stained for TNFR1 (APC; Biolegend, #113005) and MHC I (FITC; Biolegend, #116506) for 30 min at 4 °C. Cells were then fixed with 1% PFA for 1 h at 4 °C and resuspended in FACS buffer for analysis with a CytoFLEX flow cytometer (Beckman-Coulter). Data were analyzed using the CytExpert software (Beckman-Coulter).

### 2.5. Next Generation RNAseq

*OPCs*. RNAseq was performed on primary mouse WT and TNFR2^−/−^ OPCs exposed to a cytokine cocktail or PBS vehicle (GEO # pending). After extraction with Arcturus PicoPure RNA Isolation Kit (Cat# KIT0204, Applied Biosystems), RNA was further purified of residual genomic DNA by on-column digestion with RNase-free DNase (Cat# 79254, Qiagen). Library preparation and sequencing were performed at the Beijing Genomics Institute (BGI) on a BGISeq-500 platform, and 30 million, paired-end 100 base pair reads were generated. Reads were trimmed using Trim Galore v0.6.4 and aligned to the mouse genome with the Ensembl version 81 of GRCm38 as reference for annotation. The number of read pairs aligned to each gene was calculated using STAR, with read-pair alignment rates resulting between 90 and 92 percent. Differential gene expression analysis was performed using EdgeR, with normalized gene expression levels represented as fragments per kilobase per million mapped reads (FPKM) [22]. Differentially expressed genes were determined to have an adjusted *p*-value less than or equal to 0.05. For DAVID pathway analysis, Cytoscape network analysis, heatmap, and UpSet plot generation, only genes with log2CPM ≥ 1 and log2|FC| ≥ 0.585 were included.

*Microglia.* RNAseq was performed on microglia FACS-sorted from mouse spinal cords of naïve mice (GEO # pending) or mice induced with experimental autoimmune encephalomyelitis (EAE) and collected at 17 days post-induction (dpi) (previously published, GEO: GSE78082 [23]). Cells were sorted with a FACSAria instrument (BD Biosciences) based on CD45^low^CD11b^+^ labeling, collected in lysis buffer, and processed for RNA extraction using the SMART-Seq V4 UltraTM Low Input RNA Kit (Clontech Laboratories), according to the manufacturer’s protocol. Library preparation and RNA sequencing were performed at the John P. Hussman Institute for Human Genomics (Miller School of Medicine, University of Miami) using an Illumina HiSeq 2500 ultra-high throughput sequencing system. Paired-end, 125 base pairs were generated and analyzed as previously published [23].

### 2.6. Ligand–Receptor Interaction Analysis

To infer potential interactions between ligand-bearing OPCs and receptor-bearing microglia, we analyzed OPC versus microglia RNAseq datasets using a bioinformatics method adapted from CellPhoneDB [24] and a published reference list of ligand–receptor pairs [25]. We measured cell–cell interaction by defining a ligand–receptor score as the mean of the average log-FPKM of the receptor gene and the ligand gene in microglia/OPCs (n = 3–4 per cell type/condition). *p* values were calculated by comparing the actual interaction scores to the null distribution scores (determined by a permutation test for each ligand–receptor pair).

### 2.7. Real-Time RT-PCR 

Reverse transcription of RNA samples was performed using the High-Capacity cDNA Reverse Transcription Kit (Cat# 4368814, Applied Biosystems), according to the manufacturer’s protocol. Complementary DNA equal to 2–5 ng of initial total RNA was used as a template in each PCR reaction. Real-time PCR was performed in the QuantStudio 6 Flex Real Time PCR system (Applied Biosystems) with PowerUP SYBR Green PCR MasterMix (Applied Biosystems). Relative gene expression was calculated with the comparative Ct (ΔΔCt) method [26] after normalization to glyceraldehyde-3-phosphate dehydrogenase (GAPDH) gene expression. Primers for gene amplification are listed in Table S1.

### 2.8. Western Blot 

Proteins were resolved by SDS-PAGE on 4–20% TGX stain-free gradient gels (Bio-Rad) then transferred onto nitrocellulose membranes. After blocking in TBS + 5% milk, membranes were probed with antibodies against pAKT (rabbit, #4060S, Cell Signaling Technologies), AKT (rabbit, 4691S, Cell Signaling Technologies), p-p65 (rabbit, #3033S, Cell Signaling Technologies), and p65 (mouse, #6956S, Cell Signaling Technologies) overnight at 4 °C. After 3 washes with PBS + 0.05% Tween (PBS-T), membranes were incubated with horseradish peroxidase (HRP)-conjugated secondary antibodies (1 h, room temperature, 1:2000, Amersham), washed 3 times with PBS-T, and incubated in Femto Maximum Sensitivity Substrate (ThermoScientific). Membranes were imaged with a ChemiDoc system (Bio-Rad), and densitometric quantification of protein expression was performed with ImageLab software (Bio-Rad). Data were normalized to β-actin and expressed as percentage ± SEM of a reference condition.

### 2.9. Cytokine Array

WT and TNFR2^−/−^ OPCs were cultured for 3DIV in 24-well plates, exposed to a cytokine cocktail for 3 h, then switched to a cytokine-free medium. After overnight incubation, the OPC medium containing released factors was collected, spun down at 300× *g* for 5 min to remove cell debris, and frozen at −80 °C until further analysis. The experiment was run on three biological replicates (three separate OPC primary cultures), each with two technical replicates/condition. The collected OPC medium (500 μL)/sample) was analyzed for cytokine content using the Proteome Profiler Mouse XL Cytokine Array (#ARY028, R&D Systems), following the manufacturer’s protocol. After overnight incubation at 4 °C, membranes were washed and exposed to biotinylated detection antibody followed by incubation with streptavidin-HRP and Femto Maximum Sensitivity Substrate (ThermoScientific). Finally, membranes were imaged with a ChemiDoc system (Bio-Rad) and densitometric quantification of protein expression was performed with HLImage++ software (https://www.wvision.com/QuickSpots.html, accessed on 30 April 2021) after normalization to preloaded controls. 

### 2.10. Statistical Analysis 

Statistical analyses were carried out with GraphPad Prism software. Details of sample size for each experiment are included in the figure legends. Western blot, RNAseq, cell counting, and real-time RT-PCR data were analyzed by two-way ANOVA followed by Sidak or Tukey tests for multiple comparisons. Cytokine array data were tested for normality with the D’Agostino & Pearson test, and then compared with Mann–Whitney or Student’s *t* tests. Data are expressed as mean ± SEM, and *p*-values equal or less than 0.05 were considered statistically significant. 

## 3. Results

### 3.1. OPCs Upregulate and Activate TNF Receptors Following Inflammatory Stimulation

We recently demonstrated that oligodendrocyte lineage cells directly participate in the neuroinflammatory response associated with experimental autoimmune encephalomyelitis (EAE), a model of MS, by releasing immunomodulatory factors [20]. Furthermore, we showed that TNFR2 acts as a suppressor of oligodendroglia-driven neuroinflammation, conferring protection in EAE. 

**Figure 1:**
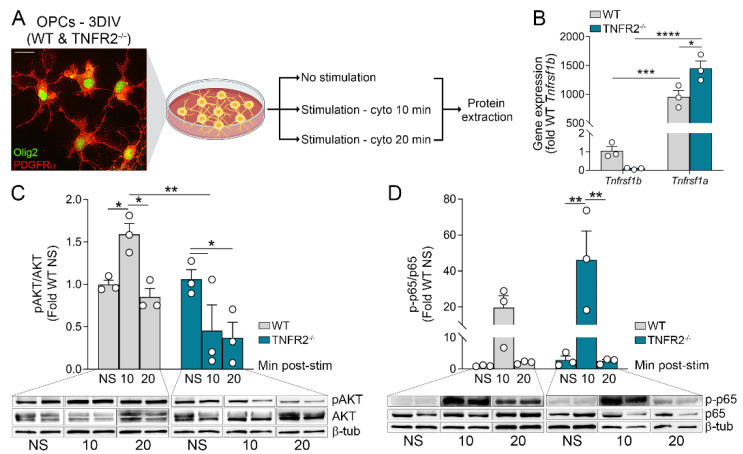
OPCs upregulate and activate TNF receptors following inflammatory stimulation. (**A**) Experimental workflow for assessing intracellular signaling activation in WT and TNFR2^−/−^ OPCs (co-expressing Olig2 and PDGFRα) following stimulation with cytokine cocktail (TNF/IFNγ/IL1β, 25 ng/mL each) at 3 days *in vitro* (DIV); scale bar: 20 μm. (**B**) Quantification of TNF receptor gene expression (*Tnfrsf1b* and *Tnfrsf1a*) in WT and TNFR2^−/−^ OPCs at 3DIV in non-stimulated conditions; n = 3, * *p* ≤ 0.05, *** *p* ≤ 0.001, **** *p* ≤ 0.0001, two-way ANOVA, Holms–Sidak multiple comparison test. (**C**) Western blot analysis of PI3K/AKT signaling activation in WT and TNFR2^−/−^ OPCs at 3DIV after 10 and 20 min of cytokine stimulation; active phosphorylated AKT (pAKT) normalized to total AKT is plotted as fold of non-stimulated (NS) WT OPCs. Results represent mean ± SEM of 3 independent experiments run in duplicate, * *p* ≤ 0.05, *** *p* ≤ 0.001, two-way ANOVA, Holms–Sidak multiple comparison test. (**D**) Western blot analysis of canonical NF-κB signaling activation PI3K/AKT signaling activation in WT and TNFR2^−/−^ OPCs at 3DIV after 10 and 20 min of cytokine stimulation; active phosphorylated p65 (p-p65) normalized to total p65 is plotted as fold of non-stimulated (NS) WT OPCs. Results represent mean ± SEM of 3 independent experiments run in duplicate, ** *p* ≤ 0.01, two-way ANOVA, Holms–Sidak multiple comparison test.

To parse out the specific contribution of OPCs in this process, we established primary cultures of OPCs from WT and TNFR2^−/−^ mice and assessed their response to an inflammatory environment by exposing them to a combination of Th1 cytokines (TNF, IL1β, and IFNγ) known to be highly upregulated in the CNS following EAE and MS (Figure 1A) [27,28]. In naïve conditions, WT OPCs expressed low levels of *Tnfrsf1b* (gene name for TNFR2) and, in comparison, markedly higher levels of *Tnfrsf1a* (gene name for TNFR1) (Figure 1B, 1.0 ± 0.2 versus 957.3 ± 109.8). Interestingly, *Tnfrsf1a* expression was significantly higher in TNFR2^−/−^ cells compared to WT (Figure 1B), possibly indicating a compensatory mechanism to maintain active TNF signaling in the absence of TNFR2. In order to verify that TNFR2-dependent intracellular signaling was inhibited as a result of TNFR2 ablation, we measured the activation of the PI3K/AKT pathway, one of the main pathways downstream of TNFR2 [8,29], by quantifying AKT phosphorylation after cytokine stimulation. In WT OPCs, phosphorylated AKT (pAKT) was significantly elevated after a 10 min stimulation and returned to basal levels at 20 min (Figure 1C). On the other hand, in TNFR2^−/−^ OPCs pAKT did not increase. In fact, it was downregulated below basal levels after both 10 and 20 min stimulation (Figure 1C). In parallel, the activation of the NF-ĸB canonical pathway, one of the main pathways downstream of TNFR1 [30], was measured by assessing phosphorylated p65 (p-p65). NF-κB was activated in both WT and TNFR2^−/−^ OPCs after 10 min stimulation and returned to baseline at 20 min. The increase in p-p65 was significantly higher in TNFR2^−/−^ compared to WT cells (Figure 1D). Notably, prolonged inflammatory stimulation (3 h with cytokines) resulted in the increase of both *Tnfrsf1a* and *Tnfrsf1b* gene expression (Figure S1A). Similar to naïve conditions, TNFR2^−/−^ cells further upregulated *Tnfrsf1a* compared to WT OPCs (Figure S1A). However, TNFR1 protein expression was not altered in a TNFR2-dependent manner when assessed by flow cytometry 18 h after stimulation (Figure S1B–D). Both the percentage of OPCs expressing TNFR1 and the mean fluorescent intensity of expression did not change due to stimulation or TNFR2 ablation (Figure S1B–D). This indicates that there is no compensatory upregulation of TNFR1 at the protein level when TNFR2 signaling is ablated and the effects observed in TNFR2^−/−^ cells cannot be attributed to enhanced TNFR1 activity.

Collectively, these data indicate that OPCs mount an innate cellular response to deal with sustained inflammation, and that TNFR2 modulates this function.

### 3.2. TNFR2 Participates in the Response of OPCs to Inflammatory Conditions

**Figure 2:**
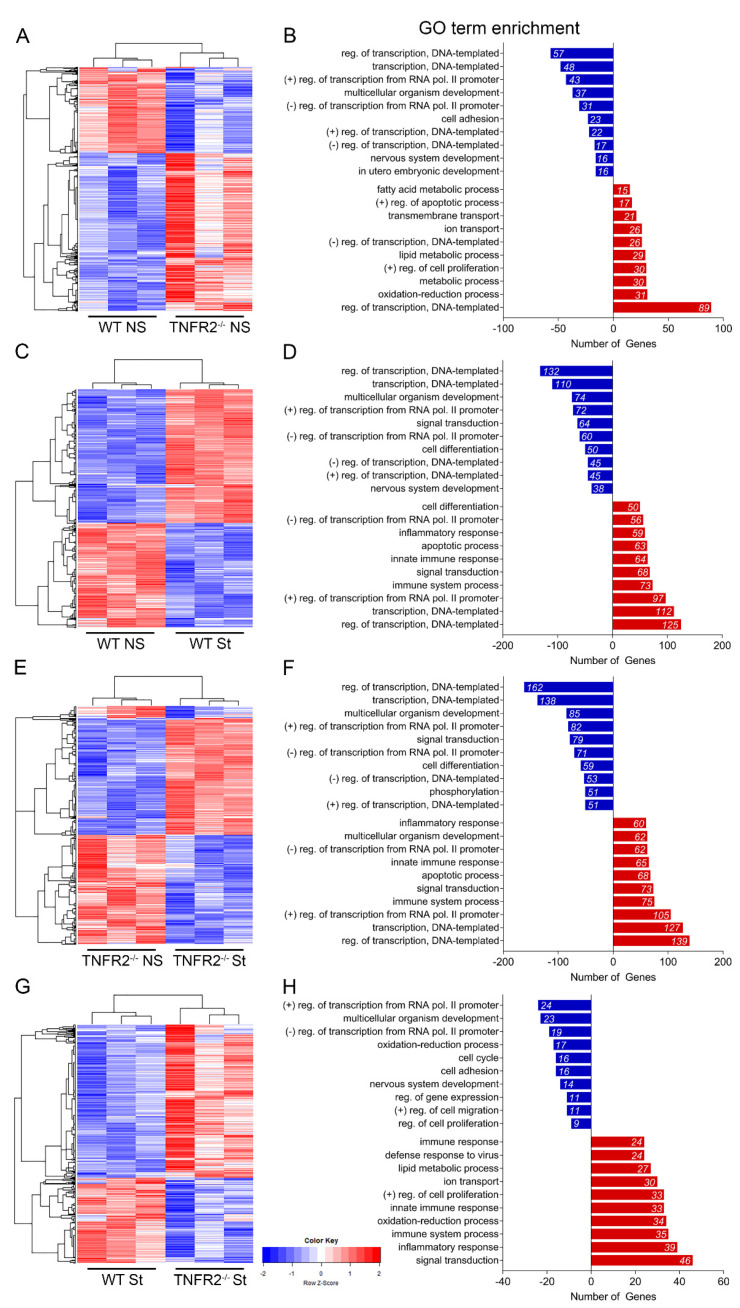
RNAseq analysis of WT and TNFR2^−/−^ OPCs *in vitro* in response to inflammatory stimuli. (**A**,**C**,**E**,**G**) Heatmap of differentially expressed genes between: non-stimulated WT and non-stimulated TNFR2^−/−^ OPCs, (**A**) stimulated WT and non-stimulated WT OPCs, (**C**) non-stimulated TNFR2^−/−^ and stimulated TNFR2^−/−^ OPCs, (**E**) stimulated WT and stimulated TNFR2^−/−^ OPCs. (**B**,**D**,**F**,**H**) Differentially expressed genes grouped by gene ontology (GO) biological process in: non-stimulated WT and non-stimulated TNFR2^−/−^ OPCs, (**B**) stimulated WT and non-stimulated WT OPCs, (**D**) non-stimulated TNFR2^−/−^ and stimulated TNFR2^−/−^ OPCs, (**F**) stimulated WT and stimulated TNFR2^−/−^ OPCs (H); blue = downregulated genes and processes; red = upregulated genes and processes; NS = non-stimulated; St = stimulated.

To determine the mechanisms by which TNFR2 signaling in OPCs regulates their response to inflammation, we analyzed by bulk RNAseq the transcriptional profile of WT and TNFR2^−/−^ OPCs in naïve conditions and after cytokine stimulation. At 3DIV, OPCs were exposed for 3 h to either a TNF/IFNγ/IL1β cocktail or vehicle, and their RNA was extracted and sequenced. Differential gene expression analysis identified several hundred upregulated and downregulated genes in each comparison. In naïve OPCs, 660 upregulated and 355 downregulated genes were found in TNFR2^−/−^ versus WT cells (Figure 2A). Gene ontology (GO) enrichment analysis showed most alterations were in genes involved in transcriptional regulation and cell metabolism (Figure 2B). Furthermore, KEGG pathway analysis determined that the downregulated genes were highly associated with the MAPK and TNF pathways, while upregulated genes were involved in metabolic functions, mainly lipid metabolism, underscoring the essential role of TNFR2 in the maintenance of OPC homeostasis (Figure S2A). As anticipated, the stimulation of OPCs resulted in large transcriptional changes compared to basal conditions. In WT OPCs, 858 upregulated and 666 downregulated genes were identified after exposure to cytokines, with transcriptional regulation and cell differentiation as the most downregulated biological processes and inflammatory response, innate immunity, and apoptosis among the most upregulated ones (Figure 2C,D). KEGG pathway analysis revealed that downregulated genes were associated with cell survival and proliferation (e.g., PI3K/AKT and cAMP signaling), while upregulated genes with inflammatory responses (e.g., TNF signaling, cytokine–cytokine receptor interaction), suggesting a shift of OPCs towards immunomodulatory function (Figure S2B). After cytokine stimulation, TNFR2^−/−^ OPCs showed upregulation of 965 genes and downregulation of 1000 genes compared to non-stimulated TNFR2^−/−^ OPCs (Figure 2E). Similar to WT cells, the stimulation of TNFR2^−/−^ OPCs resulted in downregulation of pathways and biological processes associated with cell survival (e.g., PI3K/AKT) and upregulation of those associated with inflammation (Figure 2F, Figure S2C). In the most significant comparison to dissect out TNFR2 function in inflammatory conditions (stim TNFR2^−/−^ versus stim WT), stimulated TNFR2^−/−^ OPCs showed 564 upregulated and 314 downregulated genes compared to stimulated WT cells (Figure 2G). Most of the downregulated genes were associated with cell cycle/survival (e.g., PI3K/AKT) and adhesion pathways, while the upregulated genes were associated with metabolism, immunity, and inflammation (e.g., cytokine–cytokine receptor interaction, toll like receptor signaling) (Figure 2H, Figure S2D). 

**Figure 3:**
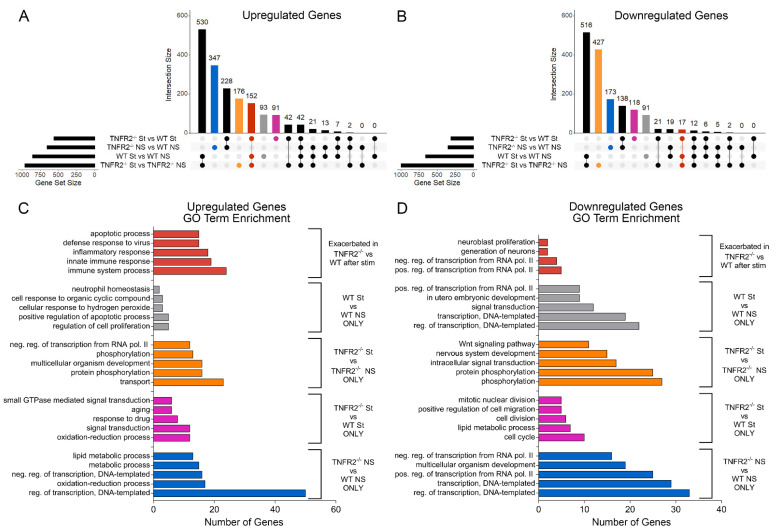
Overlap analysis of RNAseq comparisons identifies key biological processes modulated by TNFR2 signaling. (**A**,**B**) UpSet plots depicting number (horizontal black bars) and overlap (vertical bars) of (**A**) upregulated and (**B**) downregulated genes between each paired comparison; blue = differentially expressed (DE) in TNFR2^−/−^ NS versus. WT NS only; pink = DE in TNFR2^−/−^ St versus WT St only; orange = DE in TNFR2^−/−^ St versus TNFR2^−/−^ NS only; gray = DE in WT St versus WT NS only; red = DE in TNFR2^−/−^ St versus TNFR2^−/−^ NS, WT St versus WT NS, and TNFR2^−/−^ St versus WT St. (**C**) Top GO terms of upregulated genes differentially expressed in each intersection of interest. (**D**) Top GO terms of downregulated genes differentially expressed in each intersection of interest. NS = non-stimulated; St = stimulated.

To isolate genes that changed in each comparison and identify those that were dependent on TNFR2, we generated UpSet plots of upregulated (Figure 3A) and downregulated (Figure 3B) genes. This analysis showed that the inflammatory stimulation of OPCs resulted in upregulation of a set of 152 shared genes mostly associated with immune regulation (Figure 3A, red column; Figure 3C), and that 91 genes were uniquely upregulated in stimulated TNFR2^−/−^ OPCs compared to WT (Figure 3A, pink column). GO enrichment analysis established that the genes selectively upregulated in TNFR2^−/−^ versus WT stimulated OPCs were involved in signal transduction and redox processes, whereas those downregulated were associated with mitosis, cell division, and the cell cycle (Figure 3C,D). This suggests that TNFR2 signaling is important for maintaining OPC proliferation, a function that is challenged by inflammatory stimulation. 

Collectively, these data underscore that TNFR2 signaling in OPCs modulate three major processes: metabolism, cell cycle, and immune-inflammatory response.

### 3.3. TNFR2 Ablation Exacerbates Inflammation-Induced Dysregulation of the Cellular Machinery That Sustains OPC Proliferation and Differentiation

**Figure 4:**
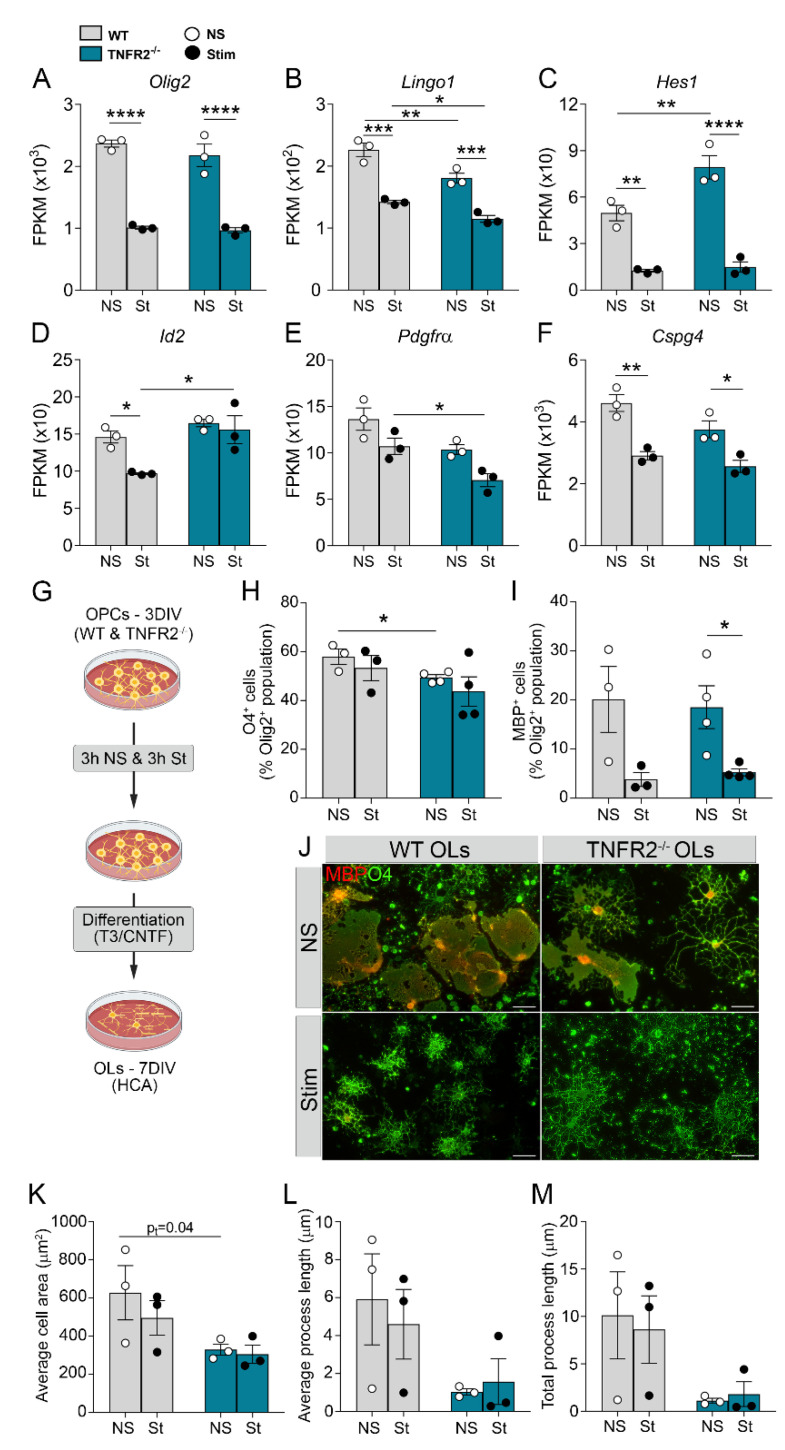
TNFR2 ablation exacerbates inflammation-induced dysregulation of the cellular machinery that sustains OPC proliferation and differentiation. (**A**–**F**) Expression profiles of select genes regulating oligodendrocyte (OL) differentiation and/or proliferation in WT and TNFR2^−/−^ OPCs following cytokine stimulation. Data, extrapolated from the RNAseq set, are expressed as fragments per kilobase per million mapped reads (FPKM). (**G**) Workflow for the OL differentiation assay. (**H**,**I**) Quantification, by high-content analysis (HCA), of differentiated O4^+^ OLs (**H**) and MBP^+^ OLs (**I**) at 7DIV. Results are expressed as a percentage of the total Olig2^+^ population. (**J**) Representative images of WT and TNFR2^−/−^ differentiated OLs at 7DIV either with or without prior stimulation with cytokines. Scale bar = 20 μm. (**K**–**M**) Quantification by HCA of (K) average cell area, (**L**) average process length, and (**M**) total process length of WT and TNFR2^−/−^ OLs. Results represent mean ± SEM of 3 independent experiments; * *p* ≤ 0.05, ** *p* ≤ 0.01, *** *p* ≤ 0.001, **** *p* ≤ 0.0001, two-way ANOVA; Holm–Sidak multiple comparison test. NS = non-stimulated; St = stimulated.

Since cell division and the cell cycle were identified as dysregulated processes when TNFR2 is ablated, we took a closer look at genes involved in OPC proliferation and differentiation in naïve and stimulated conditions. The positive regulator of differentiation *Olig2* was downregulated in both genotypes following stimulation (Figure 4A), and so were the negative regulators of differentiation *Lingo1* and *Hes1* (Figure 4B,C). In TNFR2-ablated cells, the expression of *Lingo1* and *Hes1* was reduced further, implicating TNFR2 in their transcriptional regulation, at least in part. Interestingly, the negative regulator of differentiation *Id2*, which was reduced in WT OPCs after stimulation, did not change in TNFR2^−/−^ OPCs, suggesting that, physiologically, TNFR2 may be contributing to OPC differentiation by suppressing *Id2* (Figure 4D, Figure S4A). As far as genes associated with OPC proliferation, we found *Pdgfrα* to be downregulated in TNFR2^−/−^ versus WT OPCs (Figure 4E), whereas *Cspg4* was equally reduced in cells with or without TNFR2 (Figure 4F). Collectively, these data indicate that inflammatory stimulation alters those signals important in directing OPCs towards maturation and that TNFR2 participates in their regulation. In addition, lack of TNFR2 signaling altered the expression of genes associated with cell metabolism, including the tricarboxylic acid (TCA) cycle (Figure S3A,B). In basal conditions, lack of TNFR2 lead to the upregulation of most genes involved in the TCA cycle, and several of those continued to be expressed at higher levels in TNFR2^−/−^ OPCs following stimulation. This suggests a role of TNFR2 in maintaining proper OPC metabolic function, which is essential for their proliferation and differentiation capacity.

To validate the RNAseq data from a functional standpoint and directly assess if TNFR2 is implicated in OPC differentiation into mature oligodendrocytes (OLs), we cultured WT and TNFR2^−/−^ OPCs, stimulated them at 3DIV with TNF/IFNγ/IL1β or vehicle, switched them to differentiating conditions and measured maturation by counting the number of O4^+^ OLs and MBP^+^ OLs (Figure 4G). Inflammatory stimulation did not affect the number of O4^+^ OLs (Figure 4H) in both genotypes but significantly reduced the number of MBP^+^ OLs in TNFR2^−/−^ cells (Figure 4I), suggesting that TNFR2 may play a role in the terminal stage of OL maturation into myelin-forming cells in conditions of CNS inflammatory stress. Notably, the number of O4^+^ OLs was reduced in TNFR2^−/−^ compared to WT cells without cytokine stimulation (Figure 4H), suggesting that TNFR2 may be involved in directing OPC towards differentiation during development. Morphologically, TNFR2^−/−^ OLs showed clear differences compared to WT OLs (Figure 4J). In non-stimulated conditions, TNFR2^−/−^ OLs had a more ramified appearance with less extensive MBP^+^ networks compared to WT cells. After stimulation, both TNFR2^−/−^ and WT OLs showed a dramatic reduction in the formation of MBP^+^ networks, but with TNFR2^−/−^ OLs displaying thinner, longer processes than WT cells, which featured a more compact structure with shorter bushy processes (Figure 4J). Quantification of these morphological features by high-content analysis showed a significant reduction in cell area in the TNFR2^−/−^ OLs (Figure 4K). In addition, TNFR2^−/−^ OLs showed trends towards reduced average and total process length (Figure 4L,M). This aligns with the idea that TNFR2^−/−^ cells have impaired myelin-forming capacity and repair ability in conditions of inflammatory CNS stress.

### 3.4. TNFR2 Ablation Enhances the Immunomodulatory and Inflammatory Function of OPCs in Response to Inflammatory Stimulation

**Figure 5:**
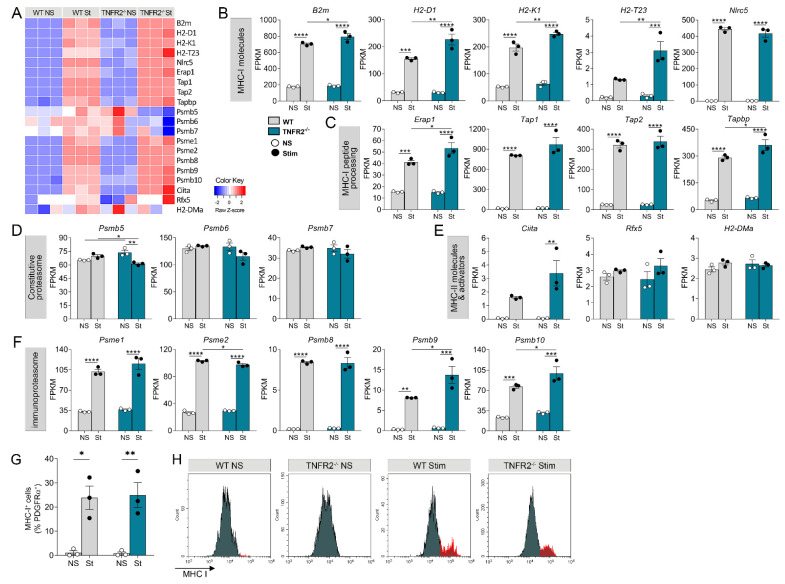
TNFR2 ablation enhances the antigen presenting function of OPCs in response to inflammatory stimulation. (**A**) Heatmap of genes related to antigen presentation expressed by WT and TNFR2^−/−^ OPCs *in vitro* with or without cytokine stimulation. (**B**–**F**) Expression profiles of genes regulating MHC-I molecules (**B**), MHC-I peptide processing (**C**,**D**) constitutive proteasome, (**D**) MHC-II molecules and activators, (**E**) and immunoproteasome (**F**). Data, extrapolated from the RNAseq set, are expressed as FPKM. (**G**) Flow cytometric quantification of MHC-I-expressing OPCs (MHC-I^+^ cells in the PDGFRα^+^ population) assessed 18 h after cells were exposed to cytokines for 3 h. (**H**) Representative flow cytometry plots of MHC-I expression in OPCs. Results represent average ± SEM of 3 independent experiments, * *p* ≤ 0.05, ** *p* ≤ 0.01, *** *p* ≤ 0.001, **** *p* ≤ 0.0001, two-way ANOVA; Holm–Sidak multiple comparison test. NS = non-stimulated; St or Stim = stimulated.

To better understand the mechanisms by which OPCs participate in immunomodulation and the role played by TNFR2 signaling in this function, we interrogated our RNAseq data sets for genes known to be implicated in various aspects of immune cell function, including antigen presentation, immune cell migration, and activation (Figure 5A). Genes important for antigen presentation by MHC-I molecules were markedly upregulated following cytokine stimulation, and several of those were further increased as a result of TNFR2 ablation, specifically *B2m*, *H2-D1*, *H2-K1*, *H2-T23*, *Erap1*, and *Tapbp* (Figure 5B,C). Components of the immunoproteasome, also involved in MHC-I-dependent antigen presentation, were upregulated (Figure 5F) as well, with *Psme2*, *Psmb9*, and *Psmb10* showing mild differential changes due to TNFR2 ablation (Figure 5D). Notably, subunits of the constitutive proteasome did not change in WT OPCs after cytokine stimulation (Figure 5D). In TNFR2^−/−^ cells, however, the *Psmb5* subunit was significantly downregulated and others (*Psmb6* and *Psmb7*) showed a downward trend (Figure 5D). Molecules associated with MHC-II-dependent antigen presentation were only minimally expressed in both WT and TNFR2^−/−^ OPCs, with no significant upregulation after cytokine stimulation with the exception of the MHC-II activator *Ciita*, which significantly increased in TNFR2^−/−^ cells (Figure 5E). Assessment of MHC-I expression in OPCs by flow cytometry showed an increase in the percent of MHC-I^+^ cells in both WT and TNFR2^−/−^ OPCs after stimulation (Figure 5G,H). However, this increase was not TNFR2-dependent at this timepoint. 

**Figure 6:**
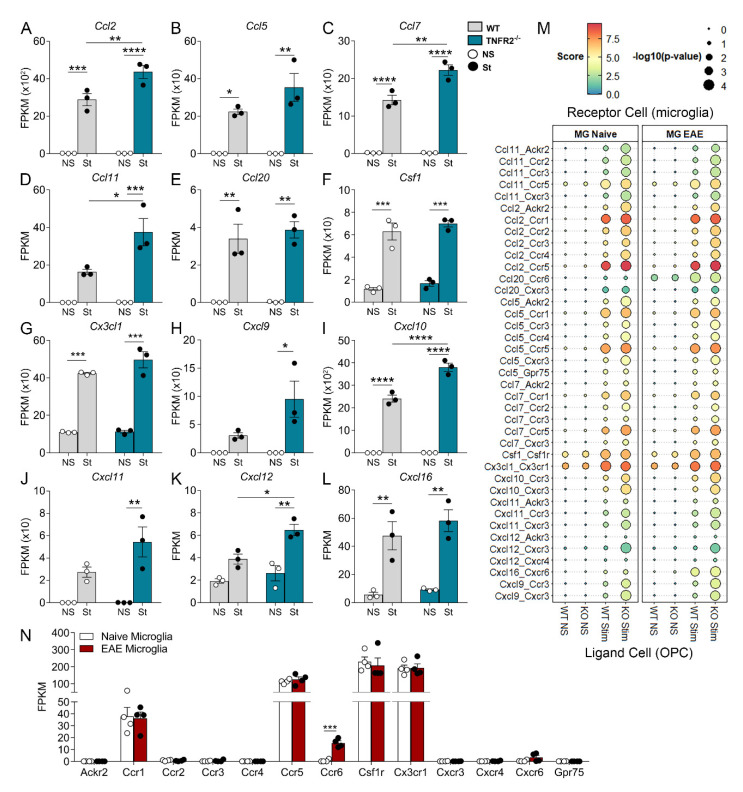
TNFR2 ablation enhances the inflammatory and immunomodulatory function of OPCs in response to inflammatory stimulation. (**A**–**L**) Expression profiles of select immunomodulatory and inflammatory genes in WT and TNFR2^−/−^ OPCs following cytokine stimulation. Data, extrapolated from the RNAseq set, are expressed as FPKM. (**M**) Dot plot of ligand–receptor interaction scores for immunomodulatory ligands expressed in OPCs and their corresponding receptors expressed in microglia in naïve conditions or at acute EAE. (**N**) Gene expression profiles of select receptors expressed in microglia *in vivo* under naïve conditions or at acute EAE and known to interact with chemokines and cytokines found to be expressed in cultured OPCs after cytokine stimulation. Results represent average ± SEM of 3 independent experiments, * *p* ≤ 0.05, ** *p* ≤ 0.01, *** *p* ≤ 0.001, **** *p* ≤ 0.0001, two-way ANOVA; Holm–Sidak multiple comparison test. NS = non-stimulated; St = stimulated.

Numerous members of the chemokine group of molecules, which are responsible for the trafficking of immune cells across the blood–brain barrier (BBB), as well as microglia migration, were significantly upregulated in both WT and TNFR2^−/−^ OPCs following cytokine stimulation (Figure 6A–K, Figure S4B). Specifically, *Ccl2*, *Ccl7*, *Ccl11*, *Cxcl10*, and *Cxcl12* were significantly more elevated in TNFR2^−/−^ compared to WT OPCs, and *Cxcl9* and *Cxcl11* were significantly increased only in TNFR2^−/−^ OPCs, indicating that TNFR2 signaling plays a role in their regulation. Notably, *Csf1*, a growth factor essential for microglia survival and proliferation, was markedly increased in OPCs of both genotypes exposed to cytokines (Figure 6L). 

To further investigate whether the chemotactic and growth factors expressed by OPCs could potentially participate in modulating the microglial response to inflammation, we took a bioinformatics approach and conducted a ligand–receptor interaction analysis comparing ligands found in our OPC RNAseq datasets with gene expression data of the corresponding receptors found in microglia. We analyzed all the validated ligand–receptor pairs published by Ramilowski et al. [25]. The microglia datasets were obtained by bulk RNAseq of FACS-sorted microglia from the spinal cord of naïve and acute EAE (17 days post-induction) C67BL/6 mice, as previously described [23]. We generated interaction scores predictive of the putative interaction between ligand_OPC_-receptor_microglia_ pairs, as previously described [25] (Figure 6M). Almost all chemokines expressed by OPCs were predicted to significantly interact with chemokine receptors expressed by naïve microglia, with the strongest interaction being Ccl2_OPC_-Ccr5_microglia_. These interactions were determined to have higher significance (lower *p* value shown as bigger size circle) for ligands expressed by stimulated TNFR2^−/−^ OPCs compared to stimulated WT OPCs, suggesting that the likelihood of interaction is also higher. *Csf1* and *Cx3cl1*, which are implicated in microglia survival and proliferation, showed significant interaction with the corresponding microglial receptors independently of TNFR2, and not only in OPCs exposed to cytokines but also in naïve OPCs as well, suggesting a role of OPCs in microglia survival in homeostatic conditions. 

**Figure 7:**
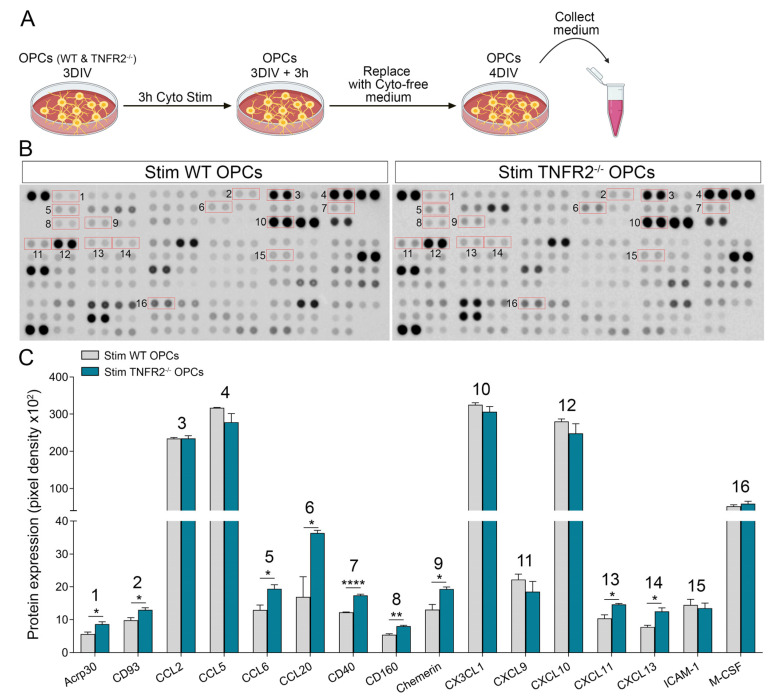
TNFR2 ablation enhances the release of immunomodulatory factors by OPCs exposed to cytokines. (**A**) Pipeline of sample preparation for cytokine array. (**B**) Representative dot blot of the cytokine array run on culture medium collected from WT and TNFR2^−/−^ OPCs following cytokine stimulation. (**C**) Densitometric quantification of soluble molecules of interest released by stimulated OPCs. Results are expressed as average pixel density ± SEM of 3 independent experiments; * *p* ≤ 0.05, ** *p* ≤ 0.01, **** *p* ≤ 0.0001, Student’s *t* test.

To directly assess the immunomodulatory molecules released by OPCs when exposed to inflammatory conditions, we analyzed the media collected from cytokine-stimulated WT and TNFR2^−/−^ OPCs (Figure 7A for experimental workflow) using a membrane-based antibody array. Stimulated OPCs were found to release a wide range of chemokines and cytokines, some at especially high levels (CCL2, CCL5, CX3CL1, and CXCL10) (Figure 7B,C; Table S2). Select molecules were significantly increased in TNFR2^−/−^ OPCs compared to WT (Acrp30, CD93, CCL6, CCL20, CD40, CD160, chemerin, CXCL11, and CXCL13), suggesting that TNFR2 signaling plays a role in suppressing inflammation by controlling these molecules (Figure 7C). Notably, various chemokines that were differentially expressed in our RNAseq analysis, such as *Ccl2* and *Cxcl10*, were not found to be different in this assay. This discrepancy could be due to the fact that the culture medium was collected approximately 1 day after stimulation, which may have led to maximal accumulation of the factors. It is likely that, to unmask differences in release between WT and TNFR2^−/−^ OPCs, the analysis needs to be performed at shorter time points after stimulation.

Overall, our data underscore that OPCs produce immunomodulatory signals, in part regulated by TNFR2, capable of directly influencing microglia activation and function, providing insight into specific molecules/pathways targetable for therapeutic purposes.

## 4. Discussion

The overarching purpose of our study was to elucidate the cellular changes that OPCs undergo when exposed to inflammatory conditions to gain insight into the mechanisms that underlie CNS repair failure in MS. Specifically, we sought to investigate the role played by TNFR2 in this process, based on previous evidence from our group indicating that TNFR2 acts as a suppressor of oligodendroglia-dependent inflammation and, at least *in vitro*, as a signal for oligodendrocyte differentiation [20,21].

We took an *in vitro* approach, stimulating primary OPC cultures from WT and TNFR2^−/−^ mice with a cocktail of Th1 cytokines (TNF/IFNγ/IL1β) known to be highly present in the MS lesion environment, and analyzing their transcriptome by RNAseq. As anticipated, hundreds of genes were differentially regulated in OPCs as a result of cytokine exposure. The gene families and processes that changed the most were those related to inflammation and immunoregulation, with cytokines, chemokines, growth factors, MHC-I, and some MHC-II components mostly upregulated. In parallel, genes and processes associated with cell proliferation and differentiation were also altered, indicating that OPCs divert from their physiological proliferation/differentiation cell programming to aid and perpetuate the immune-inflammatory response. This is in line with an *in vivo* study by Falcão et al. which reported on the transition of oligodendroglia, both OPCs and OLs, to a disease-associated state at acute EAE, characterized by the expression of immune-related genes linked to IFNγ response [31]. Our *in vitro* data largely recapitulate this observation with respect to the OPC population, demonstrating that our treatment paradigm with Th1 cytokines models quite accurately the transition that OPCs undergo *in vivo*, thus can be useful to address mechanistic questions in a simplified system. Most importantly, our data directly implicate TNFR2 signaling in the modulation of the OPC state and fate during neuroinflammation. The exacerbation of OPCs’ inflammatory profile when TNFR2 is ablated points at a role for TNFR2 in suppressing the inflammatory and antigen-presenting phenotype of OPCs. This function of TNFR2 is not unique to OPCs but extends to other glial cells. Indeed, previous work from our laboratory uncovered that, at acute EAE, TNFR2-ablated microglia develop a proinflammatory phenotype with the dysregulated expression of homeostatic and host defense genes demonstrating the immunosuppressive and anti-inflammatory role of microglial TNFR2 [23]. 

The ability of cytokines such as IFNγ, soluble TNF, and IL17 to inhibit OPC differentiation into OLs has long been known [32,33,34,35], but only recently the coupling of this effect with a shift towards the acquisition of an immunoregulatory and antigen presenting phenotype has been addressed more in depth. When simultaneously exposed to the differentiating agent T3 and IFNγ *in vitro*, OPCs upregulate MHC-I molecules, which allow for antigen processing and presentation to CD8^+^ T cells, inducing their activation and cytotoxic function [36]. Furthermore, constant low exposure to IFNγ has been shown to upregulate antigen presentation genes in OLs differentiated from iPSCs of healthy controls and MS patients [37]. Our data are in line with these reports as marked upregulation of numerous MHC-I genes was observed in OPCs following Th1 cytokine stimulation. Importantly, this effect was exacerbated in cells lacking TNFR2, indicating a direct involvement of TNFR2 signaling in the antigen-presenting function of OPCs. The upregulation of MHC-I expression was confirmed at the protein level in both WT and TNFR2^−/−^ OPCs 18 h after the 3 h cytokine stimulation, although not in a TNFR2 dependent manner. It is plausible that, by this time, expression in TNFR2^−/−^ OPCs has already peaked, thus warranting assessment at earlier time points.

The upregulation of MHC-II molecules in OPC subsets has also been reported in some experimental models [31,36]. Falcão et al. demonstrated that OPCs cultured in the presence of MOG_35–55_ peptide and CD4^+^ T cells expressing the T cell receptor for MOG_35–55_ were able to induce the proliferation of naïve, memory, and activated T cells, indicating that OPCs can execute antigen presentation via MHC-II, a process especially relevant in the context of MS. In our model, MHC-II-related molecules did not significantly change after Th1 stimulation, with the only exception of the MHC-II activator *Ciita* that was upregulated specifically in TNFR2^−/−^ OPCs, once again underscoring the role of TNFR2 in regulating the antigen-presenting function of OPCs. 

Our findings demonstrate that OPC-dependent immunomodulation in inflammatory conditions is not only ascribed to the antigen-presenting role acquired by OPCs [36] but also to their expression and release of chemoattractant factors known to recruit immune cells (lymphocytes, macrophages, microglia) into the CNS and at demyelinating lesion sites in MS and MS models [38,39,40]. These include CCL2, which was identified by ligand–receptor interaction analysis to be the chemokine released by OPCs with the highest probability to bind to CCR5-expressing microglial cells, thus promoting their migration. Importantly, this interaction reached the highest significance between stimulated OPCs lacking TNFR2 and microglia. Furthermore, since CCR5 is also highly expressed by T cells found in active MS lesions [3,41,42,43,44], it is plausible that OPC-released CCL2 may contribute to their trafficking as well. In addition to migration, TNFR2 signaling in OPCs may regulate the activation state of T cells via the co-stimulatory receptors CD40 and CD160, both of which were found to be released at higher levels by TNFR2^−/−^ OPCs compared to WT after cytokine stimulation. Indeed, CD40, a member of the TNF receptor superfamily, is known to bind to CD40ligand on T helper cells, leading to T cell activation and the exacerbation of their inflammatory properties [45,46,47], and CD160 binds to MHC-I molecules and promotes IFNγ release [48]. 

As OPCs transition to an immunomodulatory phenotype after inflammatory exposure, they dysregulate their cell proliferation and differentiation machinery, with consequent impairment of their ability to efficiently progress towards more mature states. Lack of TNFR2 worsens this outcome, as it reduces differentiation into O4^+^ cells in basal conditions and into myelin-forming MBP^+^ OLs after cytokine stimulation. This latter effect is accompanied by measurable phenotypic alterations (reduced cell area). This observation correlates with the GO enrichment analysis that shows the downregulation of genes belonging to lipid metabolic processes in stimulated TNFR2^−/−^ versus WT cells after stimulation. 

Notably, we found that several hundred genes were differentially regulated in OPCs without inflammatory stimulation and only in association with TNFR2 deletion. Considering that, by ours and others’ accounts, TNFR2 baseline expression in OPCs is low [49], this demonstrates the key role that the TNFR2 signaling pathway plays in regulating OPC function in normal physiology. Indeed, lack of this minimal TNFR2 expression is sufficient to disrupt OPCs, which behave as if they are in a status of enhanced stress. Cell metabolism is affected, as shown by the upregulation of virtually all TCA cycle genes, among other metabolic pathways. This response is typically observed in stress conditions, where the cell is pushed towards potentiating ATP production to meet injury-induced energy demands [50,51,52]. Although meant to be protective, this mechanism also leads to the accumulation of reactive oxygen species that may instead exacerbate damage [51,53,54].

The specific intracellular signaling cascades through which TNFR2 in OPCs exerts its protective functions are yet to be elucidated, but the PI3K/AKT pathway is likely to be involved, given it is one of the main signals downstream of TNFR2, and we show it to be inhibited in our TNFR2^−/−^ OPCs. That TNFR2 could be protective by balancing detrimental TNFR1 seems unlikely, since the ablation of TNFR2 does not alter TNFR1 protein levels at the membrane surface, despite upregulating its gene expression. 

More broadly, our study contributes to elucidating the role of TNF signaling in neuroinflammatory and demyelinating disease, which has been controversial mainly in light of the failure of anti-TNF drugs in MS therapy. Indeed, the only clinical trial with a non-selective TNF inhibitor, Lenercept, had to be terminated due to worsening of the disease, with increased frequency and severity of attacks and demyelinating lesions [55]. This could be explained after later studies uncovered the dichotomous function of the two forms of solTNF and tmTNF, the first promoting neurotoxic effects, the latter neuroprotective. This underscored how pan-TNF inhibition is not a viable therapeutic option, but selective targeting of solTNF-TNFR1 signaling (inhibition) and tmTNF-TNFR2 signaling (activation) should be pursued. Our study supports the idea that promoting TNFR2 signaling in the CNS has therapeutic potential. Indeed, we show that activation of this pathway in OPCs during neuroinflammation can redirect OPCs towards normal homeostasis, suppressing their pro-inflammatory shift.

## 5. Conclusions

The picture that emerges from our studies is that OPCs respond to stress by diverting their physiological functions towards inflammatory activation and immunomodulation, becoming less equipped to perform reparative functions such as remyelination. Most importantly, we demonstrate that TNFR2 plays a key beneficial role in keeping OPCs from straying from their normal physiology, as demonstrated by the fact that, in naïve conditions, TNFR2 absence alone causes enhanced cell stress. TNFR2 is even more important when OPCs are exposed to inflammatory cues, as in MS or other neuroinflammatory conditions, because its activation serves to counteract the environmental pull towards detrimental immune-inflammatory activation and to maintain OPC repair capacity. This would imply that areas where OPCs express higher levels of TNFR2 should be associated with reduced damage in neuroinflammatory diseases. This concept is indeed supported by evidence in MS brain tissue wherein higher expression of TNFR2 correlated with less severe pathology and the absence of meningeal inflammation [56].

Ultimately, besides confirming the beneficial function of TNFR2 in OPCs, our transcriptomic analysis provides an invaluable resource for the identification of molecules downstream of TNFR2 that could be targeted to promote CNS repair, which, to this day, remains a largely unmet need.

## Data Availability

All RNAseq datasets are publicly available in the NIH Gene Expression Omnibus (GEO) repository, accession numbers GEO:GSE78082 and GEO:pending.

## Supplementary Materials

The following are available online at https://www.mdpi.com/article/10.3390/cells10071785/s1: Figure S1: Gene expression of *Tnfrsf1b* and *Tnfrsf1a* is upregulated in OPCs following cytokine stimulation. Figure S2: KEGG pathway enrichment analysis shows TNFR2-dependent changes in OPCs due to cytokine stimulation. Figure S3: The tricarboxylic acid (TCA) cycle is altered in a TNFR2-dependent manner in both basal and stimulated conditions. Figure S4: Validation of RNAseq results. Table S1: Primer sequences for real time RT-PCR. Table S2: Immunomodulatory factors released by WT and TNFR2^−/−^ OPCs after inflammatory stimulation.
