# Supplementary material for: TNFR2 Signaling Regulates the Immunomodulatory Function of Oligodendrocyte Precursor Cells
Source: Cells. 2021 Jul 15;10(7):1785. doi: 10.3390/cells10071785 (PMC8306473; doi:10.3390/cells10071785)
Supplement: Supplementary file 1 [file cells-10-01785-s001.zip › cells-1223293-supplementary.pdf]

Supplementary Materials

TNFR2 Signaling Regulates the Immunomodulatory Function of Oligodendrocyte Precursor Cells

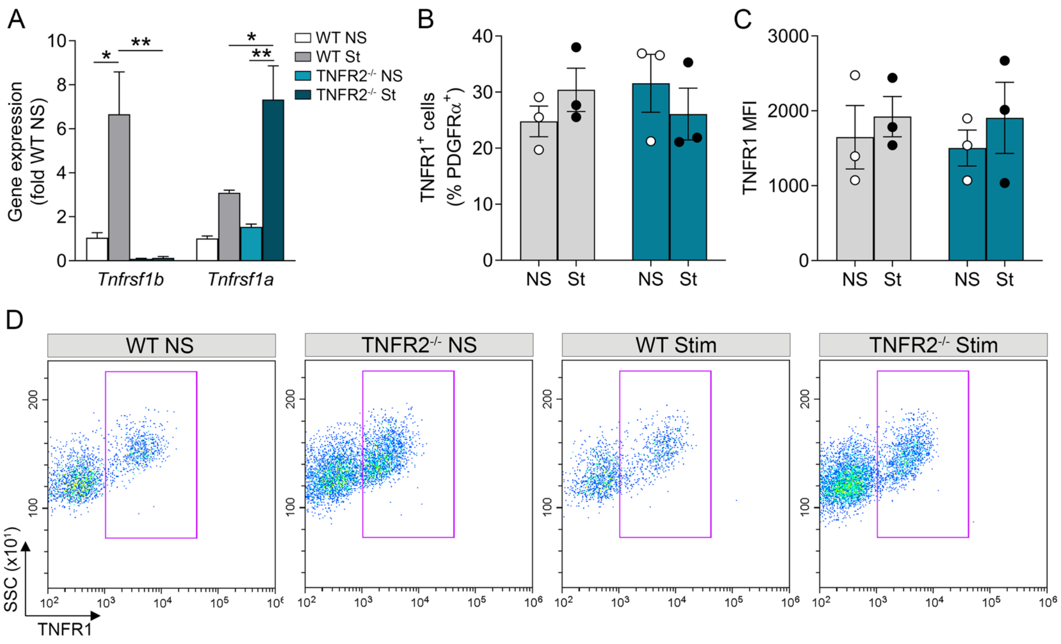

Figure S1. Gene expression of *Tnfrsf1b* and *Tnfrsf1a* is upregulated in OPCs following cytokine stimulation. (A) Gene expression quantification of *Tnfrsf1b* and *Tnfrsf1a* by real-time RT-PCR. (B-C) Flow cytometric quantification of TNFR1 protein expression in WT and TNFR2-/- OPCs 18h after a 3h cytokine stimulation; number of TNFR1+ OPCs as percentage of PDGFRα+ cells (B) and mean fluorescent intensity (MFI) of TNFR1 expression in OPCs (C) are shown. (D) Representative flow cytometry plots of TNFR1 expression. Results represent average ± SEM of 3 independent experiments; *p≤0.05, ** p≤0.01, ****p≤0.0001, two-way ANOVA; Holm-Sidak multiple comparison test. NS = non-stimulated; St = Stimulated.

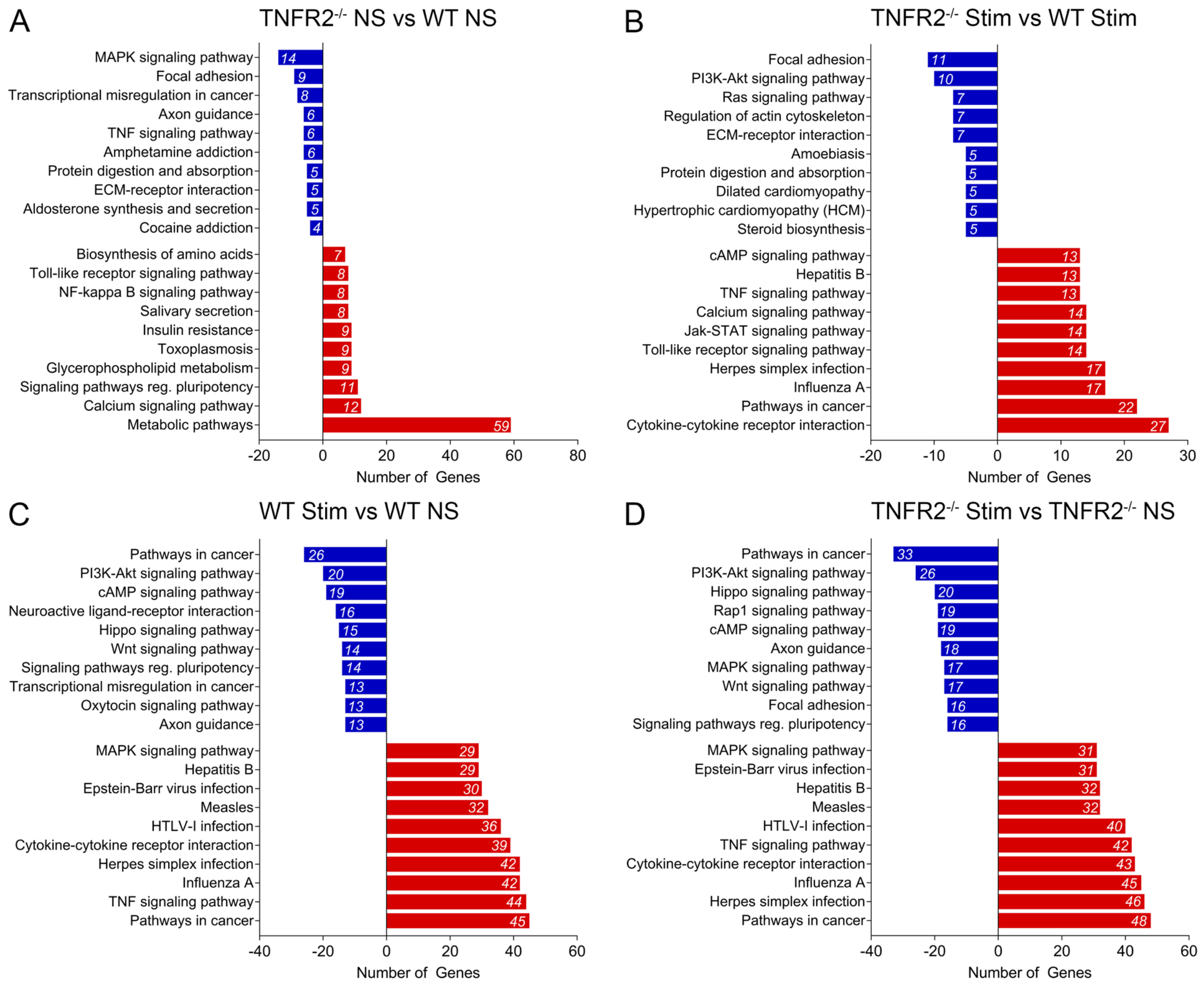

Figure S2. KEGG pathway enrichment analysis shows changes in OPCs due to cytokine stimulation, and partly regulated by TNFR2. KEGG pathway analysis of (A) TNFR2-/- NS vs WT NS, (B) TNFR2-/- Stim vs WT Stim, (C) WT Stim vs WT NS, (D) TNFR2-/- Stim vs TNFR2-/- NS. Blue = downregulated pathways/processes, Red = upregulated pathways/processes.

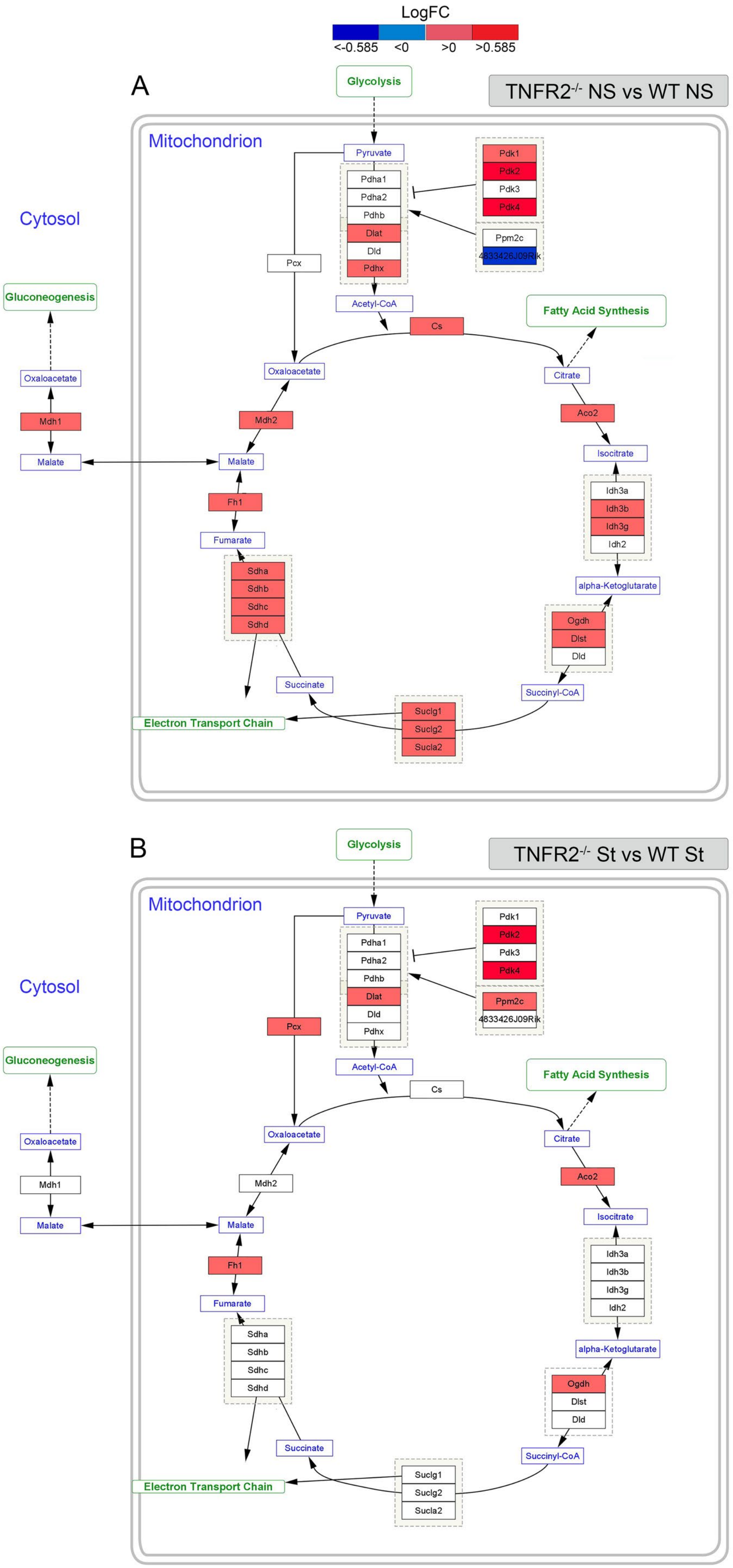
LogFC
<-0.585
<0
>0
>0.585
A
TNFR2-/- NS vs WT NS
Glycolysis
Mitochondrion
Cytosol
Pyruvate
Pdha1
Pdha2
Pdhb
Dlat
Dld
Pdhx
Pdk1
Pdk2
Pdk3
Pdk4
Ppm2c
4833426J09Rik
Pcx
Acetyl-CoA
Cs
Fatty Acid Synthesis
Gluconeogenesis
Oxaloacetate
Oxaloacetate
Citrate
Mdh1
Mdh2
Aco2
Malate
Malate
Isocitrate
Idh3a
Idh3b
Idh3g
Idh2
Fh1
Fumarate
alpha-Ketoglutarate
Sdha
Sdhb
Sdhc
Sdhd
Ogdh
Dlst
Dld
Succinate
Succinyl-CoA
Electron Transport Chain
Suclg1
Suclg2
Sucla2
B
TNFR2-/- St vs WT St
Glycolysis
Mitochondrion
Cytosol
Pyruvate
Pdha1
Pdha2
Pdhb
Dlat
Dld
Pdhx
Pdk1
Pdk2
Pdk3
Pdk4
Ppm2c
4833426J09Rik
Pcx
Acetyl-CoA
Cs
Fatty Acid Synthesis
Gluconeogenesis
Oxaloacetate
Oxaloacetate
Citrate
Mdh1
Mdh2
Aco2
Malate
Malate
Isocitrate
Idh3a
Idh3b
Idh3g
Idh2
Fh1
Fumarate
alpha-Ketoglutarate
Sdha
Sdhb
Sdhc
Sdhd
Ogdh
Dlst
Dld
Succinate
Succinyl-CoA
Electron Transport Chain
Suclg1
Suclg2
Sucla2

Figure S3. The tricarboxylic acid (TCA) cycle is altered in a TNFR2 dependent manner in both basal and stimulated conditions. (A-B) Cytoscape pathway network analysis of TNFR2 dependent differentially expressed genes in the TCA cycle for both (A) non-stimulated and (B) cytokine stimulated conditions. NS = non-stimulated; St = Stimulated. Blue = downregulated genes, Red = upregulated genes.

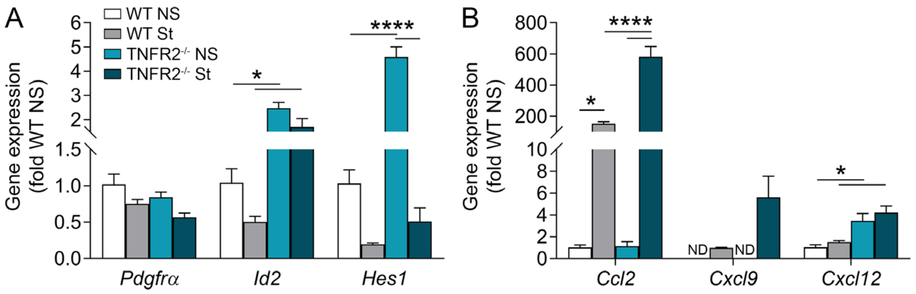

Figure S4. Validation of RNAseq results. Gene expression quantification by real-time RT-PCR of select genes associated with (A) OPC proliferation/differentiation, (B) inflammatory response. Results represent average ± SEM of 3 independent experiments, *$p \leq 0.05$, ** $p \leq 0.01$, ****$p \leq 0.0001$, two-way ANOVA, Holm-Sidak multiple comparison test. NS = non-stimulated; St = Stimulated.

Table S1. Primer sequences for real time RT-PCR.

Gene Name	Primer Sequence
Tnfrsf1b	F: 5′ acacagtgcccgcccaggttg 3′<br>R: 5′ ggccaggaggacacttagcacagc 3′
Tnfrsf1a	F: 5′ gcccgaagtctactccatcatttg 3′<br>R: 5′ ggctggggagggggctggagttag 3′
Pdgfrα	F: 5′ cctggcatgatggtcgattctact 3′<br>R: 5′ ggtctcttctcgggctcactgttc 3′
Id2	F: 5′ cccagcatcccccagaacaaga 3′<br>R: 5′ tggtgatgcaggctgacgatagtg 3′
Hes1	F: 5′ tccaagctagagaaggcagacatt 3′<br>R: 5′ cggcgcggtatttccccaacac 3′
Ccl2	F: 5′ ccccactcacctgctgctac 3′<br>R: 5′ cctgctgctggtgattctctt 3′
Cxcl9	F: 5′ agtccgctgttcttttcctttg 3′<br>R: 5′ gtgcctcggctggtgctgat 3′
Cxcl12	F: 5′ gagagccacatcgccagagc 3′<br>R: 5′ ggatccactttaatttcgggtcaa 3′
Gapdh	F: 5′ gaggccggtgctgagtatgtcgtg 3′<br>R: 5′ tcggcagaaggggcggagatga 3′

Table S2. Immunomodulatory factors released by WT and TNFR2$^{-/-}$ OPCs after inflammatory stimulation.

PROTEIN	WT St (APD ± SEM)	TNFR2$^{-/-}$ St (APD ± SEM)	TNFR2$^{-/-}$ St vs WT St (fold change)	*p* value
Adiponectin	554.3 ± 64.4	860.1 ± 71.7	1.5515	0.0338
Amphiregulin	819.4 ± 149.1	1066.7 ± 102.6	1.3018	0.2437
Angiopoietin-1	879.7 ± 121	1147.2 ± 115.7	1.3041	0.1855
Angiopoietin-2	856 ± 108.9	932.9 ± 173.6	1.0899	0.7264
Angiopoietin-like 3	1309.2 ± 117.2	1570.8 ± 94.3	1.1998	0.157
C1qR1 [CD93]	977.1 ± 81.4	1298.2 ± 64.8	1.3287	0.0367
CCL11	2299.9 ± 43.2	2223.4 ± 485.1	-1.0344	0.8828
CCL12	5593.6 ± 755.2	5177.8 ± 3087.6	-1.0803	0.9022
CCL17	1475.8 ± 105	1437.6 ± 157.6	-1.0266	0.8499
CCL19	1133.5 ± 109.6	1399 ± 151.5	1.2343	0.2287
CCL2	23434.1 ± 291.3	23458.9 ± 761.8	1.0011	0.9772
CCL20	1688.3 ± 619.8	3637.8 ± 77.8	2.1547	0.0355
CCL21	815.7 ± 99.5	1106.2 ± 82.3	1.3561	0.0877
CCL22	1742.4 ± 89	1975.9 ± 62	1.134	0.0978
CCL3/CCL4	1645.6 ± 119.1	1613.7 ± 63.8	-1.0198	0.8251
CCL5	31685.1 ± 130.4	27857.7 ± 2271.4	-1.1374	0.1678
CCL6	1293.7 ± 146.6	1938.5 ± 124.5	1.4984	0.0285
CD14	1795.1 ± 142.6	2005 ± 49.2	1.1169	0.2364
CD160	534.7 ± 28.9	798.6 ± 25.8	1.4937	0.0024
Chemerin	1303.7 ± 154.9	1930.4 ± 64.2	1.4806	0.0202
Chitinase 3-like 1	845.1 ± 97.8	3055.2 ± 1181.4	3.6154	0.1357
Coagulation Factor III/ Tissue Factor	1925.7 ± 161	2235.5 ± 308.4	1.1609	0.4235
Complement Component C5/C5a	590.6 ± 103.9	886.9 ± 157.9	1.5017	0.1921
Complement Factor D	484 ± 170.1	696.5 ± 84.3	1.439	0.3256
C-Reactive Protein	-14.7 ± 96.5	63.7 ± 21.6	-4.3302	0.4727
CX3CL1	32523.9 ± 550.7	30638.7 ± 1406.3	-1.0615	0.28
CXCL1	35907.7 ± 1123.4	38446.2 ± 529.3	1.0707	0.1104
CXCL10	28078.8 ± 681.2	24824.2 ± 2632.7	-1.1311	0.2975
CXCL11	1035.8 ± 111.3	1457.9 ± 32.6	1.4074	0.022
CXCL13	773.2 ± 50.2	1252.1 ± 106.8	1.6194	0.0154
CXCL16	2354 ± 296.5	1884.7 ± 94.2	-1.249	0.2059
CXCL2	11848.1 ± 663.5	9639.2 ± 2354.8	-1.2292	0.4176
CXCL9	2221 ± 162.9	1849.6 ± 315.5	-1.2008	0.3546
Cystatin C	17720.5 ± 472.7	20080.6 ± 1186.1	1.1332	0.1383
DKK-1	1011.5 ± 160.9	1400.2 ± 91.3	1.3843	0.1035
DPPIV [CD26]	798.8 ± 75.3	1221.5 ± 146.5	1.5291	0.0623
EGF	980.6 ± 105.1	1262.7 ± 76.7	1.2877	0.096
Endoglin [CD105]	459.4 ± 76.4	686.1 ± 109.2	1.4936	0.1641
Endostatin	1717.1 ± 413.7	2256.5 ± 583.8	1.3141	0.4929
E-Selectin [CD62E]	1095.3 ± 127.7	1330.4 ± 216.2	1.2146	0.4021
Fetuin A	1900.5 ± 196	2308.4 ± 281	1.2146	0.2996
FGF-21	1339.1 ± 150.5	1512.5 ± 210.9	1.1294	0.5401
FGFa	867.8 ± 47.7	1249.4 ± 151	1.4399	0.0736

Flt-3 Ligand	830.7 ± 63.6	976.7 ± 60.8	1.1758	0.1724
Gas 6	1239.6 ± 189.4	1566.6 ± 99.7	1.2638	0.2014
G-CSF	1054.4 ± 100	1079.3 ± 106.4	1.0236	0.873
GDF15	1225.1 ± 144.6	1471.4 ± 143	1.2011	0.2925
GMCSF	534.3 ± 97.5	993 ± 259.5	1.8586	0.1734
HGF	766.3 ± 71.2	898.8 ± 100.2	1.173	0.3417
ICAM-1 [CD54]	1439.7 ± 174.5	1349.9 ± 148.6	-1.0665	0.7152
IFNγ	1070.3 ± 121.8	1237.1 ± 78.8	1.1557	0.3146
IGFBP1	1266.6 ± 78	1654.8 ± 258.2	1.3065	0.2235
IGFBP2	33894.3 ± 1140.6	35229 ± 1090	1.0394	0.4452
IGFBP3	28758.3 ± 854	29999.8 ± 848.3	1.0432	0.3606
IGFBP-5	2107 ± 245.1	3496.8 ± 743.7	1.6596	0.1506
IGFBP-6	1398.3 ± 187	1785.1 ± 32.3	1.2766	0.1112
IL10	1559.5 ± 134.9	2030.9 ± 252.1	1.3023	0.1746
IL11	1658.7 ± 215.1	2364 ± 257.8	1.4252	0.1036
IL-12p40	1050.6 ± 202.7	1232.3 ± 134.4	1.173	0.4964
IL13	1106.6 ± 112.9	1498.1 ± 182.4	1.3538	0.1421
IL15	1447.4 ± 247.5	1888.7 ± 228.3	1.3049	0.2602
IL-17A	144.2 ± 61.8	203.4 ± 62.2	1.4105	0.5364
IL1α	1002.2 ± 96	1324.9 ± 163.9	1.322	0.1645
IL1β	7837.7 ± 962.7	4065.6 ± 1929	-1.9278	0.1551
IL1ra	1185.9 ± 78.2	1431.4 ± 204.4	1.207	0.3248
IL2	113.6 ± 63.2	215.6 ± 44.9	1.8975	0.2585
IL22	509 ± 75.1	642.6 ± 87.9	1.2625	0.3123
IL23	880.3 ± 134.4	1095.1 ± 150	1.244	0.3462
IL27p28	1722.7 ± 208.5	1953.6 ± 99.9	1.134	0.3744
IL-28	5171.2 ± 693.8	7145.1 ± 478.7	1.3817	0.0792
IL3	301 ± 71.8	412.2 ± 37.3	1.3692	0.2415
IL33	2721.8 ± 440.4	3435.6 ± 309	1.2623	0.2552
IL4	1212.2 ± 197.1	1640.1 ± 149.9	1.353	0.1591
IL5	1133.8 ± 157.7	1457.3 ± 121.7	1.2853	0.1797
IL6	4249.9 ± 1387.8	2364.5 ± 193.6	-1.7974	0.2497
IL7	2758.2 ± 422.2	3421.3 ± 467.8	1.2404	0.3521
LDL R	5382.6 ± 1050.6	4588.3 ± 485.4	-1.1731	0.5303
Leptin	1181.2 ± 135.2	1768.2 ± 256.7	1.497	0.1131
LIF	3314.3 ± 309	5240 ± 972.6	1.581	0.1322
Lipocalin	13020.3 ± 2331.7	17275.3 ± 674.9	1.3268	0.1545
LIX	6169.4 ± 1182.5	6525.5 ± 2994.7	1.0577	0.9173
MCSF	5190.2 ± 386.9	5864.3 ± 678	1.1299	0.4366
MMP2	2679.3 ± 206.4	3250.6 ± 269.6	1.2132	0.1678
MMP3	1364.6 ± 971.1	5225.2 ± 2523.5	3.8291	0.2265
MMP-9	269 ± 99.2	332.9 ± 142.5	1.2377	0.7314
Myeloperoxidase	1710.5 ± 377.8	2209.7 ± 379.4	1.2918	0.404
Osteopontin	13211.8 ± 4517	16326.3 ± 4729.3	1.2357	0.6588
PDECGF	577.8 ± 194.5	931.5 ± 315.8	1.6121	0.3943
PDGFBB	1724.3 ± 90.9	2117.3 ± 249.9	1.2279	0.2135

Pentraxin 2	1227 ± 91.7	1818.7 ± 234.5	1.4823	0.0785
Pentraxin 3	24075.4 ± 590	23820 ± 1149	-1.0107	0.8529
Periostin	556.9 ± 128.6	649.3 ± 159.6	1.1659	0.6755
Pref-1	1670.2 ± 181.3	1881.3 ± 182.1	1.1264	0.4575
Proliferin	1113.4 ± 162.4	1481.9 ± 225.2	1.331	0.2552
Proprotein Convertase 9	2590.6 ± 345.1	2777.2 ± 396	1.072	0.7404
P-Selectin [CD62P]	1209.7 ± 163.4	1319.1 ± 47	1.0904	0.555
RAGE	1023.9 ± 268.7	1238.5 ± 256.5	1.2096	0.5945
RBP4	641 ± 101.1	1087.3 ± 178.9	1.6962	0.0956
Reg3G	1198.4 ± 392.2	1374.5 ± 265.5	1.147	0.7288
Resistin	215.1 ± 129	393.1 ± 99.6	1.8275	0.3363
Serpin E1	2896.7 ± 507.4	3773.2 ± 888.7	1.3026	0.44
Serpin F1	1592.1 ± 178.4	1941.4 ± 171	1.2194	0.2305
Thrombopoietin	922.7 ± 70.5	1034.8 ± 159.1	1.1215	0.5547
TIM-1	1077.9 ± 108.2	1288.5 ± 236.3	1.1954	0.4631
TNF	3033.4 ± 246.9	2929.1 ± 315.8	-1.0356	0.8076
TNFRSF11B	2122.7 ± 164.7	9541.5 ± 5890.8	4.495	0.2765
TNFRSF5 [CD40]	1222.3 ± 9.7	1735.3 ± 37.4	1.4197	0.0002
TNFSF13B	1106.1 ± 95.9	1040.3 ± 207.8	-1.0632	0.7882
VCAM-1 [CD106]	4319.3 ± 545.8	3138.9 ± 205.2	-1.3761	0.1129
VEGF	1135.9 ± 189.5	1571.1 ± 339.6	1.3831	0.3259
WISP1	1589.4 ± 182.8	2344.8 ± 709	1.4753	0.3605
